# Fluidic enabled bioelectronic implants: opportunities and challenges

**DOI:** 10.1039/d2tb00942k

**Published:** 2022-08-01

**Authors:** Lawrence Coles, Pelumi W. Oluwasanya, Nuzli Karam, Christopher M. Proctor

**Affiliations:** Electrical Engineering Division, Department of Engineering, University of Cambridge Cambridge UK cmp81@cam.ac.uk

## Abstract

Bioelectronic implants are increasingly facilitating novel strategies for clinical diagnosis and treatment. The integration of fluidic technologies into such implants enables new complementary routes for sensing and therapy alongside electrical interaction. Indeed, these two technologies, electrical and fluidic, can work synergistically in a bioelectronics implant towards the fabrication of a complete therapeutic platform. In this perspective article, the leading applications of fluidic enabled bioelectronic implants are highlighted and methods of operation and material choices are discussed. Furthermore, a forward-looking perspective is offered on emerging opportunities as well as critical materials and technological challenges.

## Abstract 2

Fluidics are enabling new possibilities in bioelectronic medicine and research.

## Graphical abstract

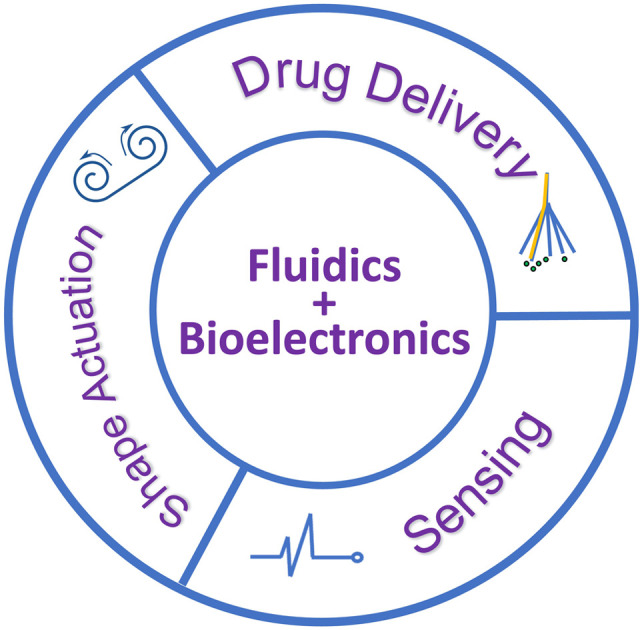

## 1 Introduction

The convergence of electronics and biology has opened new possibilities in healthcare. Bioelectronics enable direct interaction with complex biological systems, allowing further understanding of how these systems work through various sensing modalities alongside targeted therapy. Implantable bioelectronics, in particular, have become an important part of modern medicine with devices such as pacemakers, spinal cord stimulators and cochlear implants improving the lives of thousands of patients every year.^1,2^

Numerous bioelectronic technologies under development promise to expand the impact and applications of this growing field of medicine. Recent developments include thin-film flexible electrocorticography devices for neural recording and stimulation,^3–5^ wireless and bioabsorbable pacemakers for cardiac monitoring,^6^ and periphery nerve interfaces for immune system modulation.^7,8^ Likewise conducting polymers, such as PEDOT:PSS, are increasingly used instead of conventional metal electrodes to create a low impedance, high capacitance electrical interface with surrounding tissue.^9–12^ A growing body of evidence has also highlighted the importance of mechanical properties and device geometry when it comes to mitigating the foreign body response to such implants.^13^ As such bioelectronic implants are increasingly composed of soft and flexible materials to better match the mechanical properties of the surrounding tissue. Furthermore, the use of thinner materials allows for conformal coverage of the complex surfaces that characterise most parts of the body, such as the brain, skin or heart.^5,14,15^

Material advancements in bioelectronics are also increasingly enabling implants to incorporate fluidics to expand functionalities. Fluidic-based interactions with the body have long been a fundamental part of healthcare. Bodily fluids are routinely taken for diagnosis and monitoring of key biomarkers, while treatment is administered through fluidic drug infusion. As fluids drive key clinical interventions, integrating similar fluidic systems into bioelectronic devices can present unique avenues of interaction with tissues and metabolic pathways. For example, this could include bioelectronic interaction with the body at the cellular level, such as chemotherapy delivery of tumour suppressing molecules^16^ or macro-scale interaction to control organ function, *e.g.* mechanical assistance of the heart.^17^ Fluidic components can also change shape in response to local and external pressures leading to opportunities for sensing as well as shape-actuation to, for example, reduce surgical footprints. In this perspective, we briefly review the state-of-the-art in bioelectronic implants with integrated fluidic components and provide a forward-looking view on the opportunities and challenges in the field.

## 2 Drug delivery

The most prevalent application of fluidics in bioelectronics is to enable drug delivery. Multifunctional bioelectronic drug delivery devices that combine sensing and drug delivery have tremendous therapeutic potential. Such devices can use real-time information about biomarkers that can then be incorporated into algorithms that control the timing and quantity of drug delivery. Neurological disorders such as epilepsy and Parkinson's disease as well as other diseases such as diabetes^18–23^ have been treated with closed-loop drug delivery. Such devices may control drug release by a variety of means from pressure to electric-field to diffusion. Moreover, drug delivery through soft and flexible microfluidic devices offers advantages when compared with both soft material-coated and uncoated non-flexible alternatives.^24^ These desirable device properties impose stringent requirements on the fabrication process flow. In the following sections, related developments in drug delivery approaches and control systems for bioelectronics are highlighted.

### 2.1. Drug delivery approaches

#### 2.1.1. Convection enhanced delivery (CED)

**Fig. 1:**
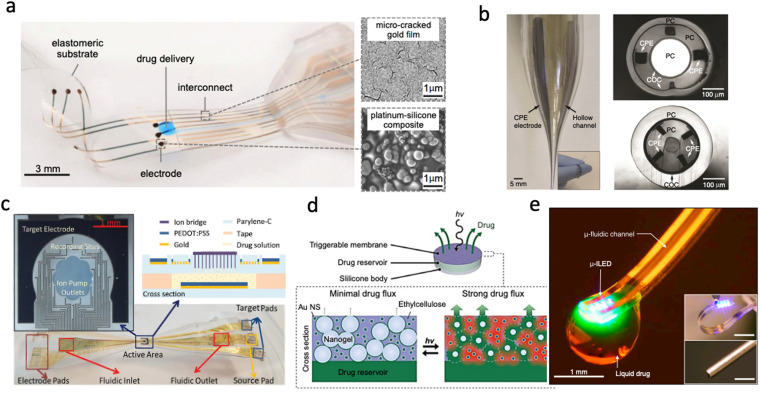
Different drug delivery devices. (a) Convection-enhanced drug delivery device based on PDMS showing the delivery outlets as well as the collocated electrodes (Reproduced with permission from AAAS.^25^ Copyright 2015 American Association for the Advancement of Science) (b) Multifunctional device capable of recording, drug delivery and light stimulation showing the optic channel, microfluidic channels and the recording sites (Reproduced with permission from Springer Nature.^26^ Copyright 2015) (c) Electrophoretic Ion Pump device showing the active area and recording sites as well as the fabricated device material stack (Reproduced with permission.^33^ Copyright 2017 John Wiley & Sons) (d) Light-activated membrane-gated drug delivery device showing how the porosity of the membrane is changed with exposure to light and the subsequent release of drugs (Reproduced with permission.^34^ Copyright 2014 National Academy of Sciences) (e) Wireless optofluidic device showing micro inorganic LEDs combined with a microfluidic channel on PDMS. (Reproduced with permission.^35^ Copyright 2015 Elsevier).

Convection enhanced delivery (CED)^25–28^ utilises an applied pressure gradient to expel drugs through outlets of (micro)fluidic channels into desired locations. Fig. 1(a) shows a CED device was integrated into a soft bioelectronic implant to enable drug delivery in a rodent model for spinal cord injury. The device was fabricated on an elastomeric substrate through a bespoke process and was shown to restore locomotion to adult rats with paralysis of both legs with minimal foreign body response.^25^ CED is very effective for delivering drugs over very large areas in a short period. It is controlled by pumps to ensure a set flow rate and volume are delivered. The disadvantages of this approach include backflow and pressure build-up caused by solution accumulation at the site of delivery. This is particularly problematic in areas such as the brain because backflow can cause the drug to reach areas not intended. Fluid build-up may also push on soft tissue to create more space which can cause lasting damage.^29,30^ Fabrication processes such as self-assembly, lamination, facile mixing, and *in situ* polymer curing, have been applied to make CED devices.^26,31^ The variety of fabrication approaches and relatively facile device design have led CED to be a widely adopted drug delivery modality in multifunctional bioelectronic implants.^32^ For example, a CED device was integrated into a multifunctional hydrogel-based platform that possesses the dual property of stiffness needed for implantation and tissue-like mechanical properties after implantation. The device's capabilities included single neuron electrophysiology recording, drug delivery and optical stimulation in freely moving mice with functionality for half a year.^21^Fig. 1(b) shows another CED device that utilised multimaterial fibre technology (thermal drawing) to create a multifunctional drug delivery, optical stimulation and neural recording platform with easy backend connections.^26^

#### 2.1.2. Electrophoretic delivery

Electrophoretic drug delivery devices^33,36–39^ use an applied electric field to deliver charged drugs on demand. One class of such devices, known as iontophoretic devices deliver ionised drugs through either open fluidic channels or microporous membranes.^40,41^ Other approaches such as organic electronic ion pumps, make use of charge-selective membranes such as polystyrene sulfonate to maximize drug delivery while limiting uptake of oppositely charged ions. The main advantage of electrophoresis is precise temporal control of the delivery rate. The ‘dry’ delivery also avoids pressure build-up at the delivery site as drug molecules are delivered with minimal solvent. The device can deliver against pressure and concentration gradient if there is a replenishment of ions. Fig. 1(c) shows a flexible electrocorticography device with drug delivery functionality. It was used to electrophoretically deliver neurotransmitters in a rodent model while simultaneously monitoring cortical activity. Drugs could also be easily switched through the microfluidic channels without unintentional delivery due to the presence of a membrane over the outlet.^33^ A similar device construct was used to demonstrate open-loop seizure control in the hippocampus of mice. Such devices are typically composed of a combination of parylene-c and SU-8 with conducting polymer electrodes. Devices are often battery powered though it has also been shown that wearable photovoltaic cells can also provide sufficient power.^36^

#### 2.1.3. Diffusion/osmosis-controlled delivery

Diffusion/osmosis removes the need for a pressure gradient (required by CED devices shown in Fig. 1(a) and (b)) by enabling the flow of drugs either from a region of higher concentration or through a semipermeable membrane, which stops when the concentration is uniform both within and outside the drug reservoir.^42–44^ The influx of water into a fixed drug reservoir over a concentration gradient/semipermeable membrane may force the ejection of drugs from the reservoir. Delivery rate, timing and volume can be difficult to control and will depend on both the diffusion coefficient and the concentration of the delivery site medium. To overcome this challenge, triggerable membranes with unique properties have been developed with sensitivity to specific wirelessly deliverable stimuli. Fig. 1d shows an implantable drug delivery device with a drug reservoir sealed by a nanocomposite (comprising gold nanoparticles in ethylcellulose matrix) membrane developed by Timko *et al.*^34^ The permeability of this membrane can be modulated by near-infrared laser irradiation. When the membrane is irradiated, the gold nanoparticles heat up which causes a reversible, ten-fold contraction of the interconnected polymer nanoparticles thus providing a pathway for drug release from the reservoir (Fig. 1d). The device showed controllable delivery over a 14 day period with pulsable functionality. Other membrane types with specific attributes can also be developed for this application.^26,45–49^ Diffusion-controlled drug delivery can be achieved from a CED device by switching the operation mode.^50^ This is however difficult to achieve without fine pressure control.

#### 2.1.4. Other delivery approaches

In some applications, drug delivery is combined with some other stimulation depending on the application. A common example is optofluidics, wherein drug delivery may be combined with light stimulation. Soft optofluidic bioelectronic devices (Fig. 1e) combine drug delivery through microfluidic channels with light stimulation to achieve pharmacology and optogenetic manipulation of neural circuitry wirelessly.^35,51,52^ However, the underlying (micro)fluidics may be controlled by any approach.

### 2.2. Control systems

Control systems consist of hardware and software, including the algorithms that trigger the release of drugs. Earlier work in this area primarily covered open-loop systems, where drug release is manually triggered upon the onset of specific conditions. Such interventions may include switching on a micropump or a power supply/electric field. The medium for delivery of this intervention is also an important consideration. For example, wireless control might be more beneficial in studies involving freely moving animals whilst tethered animal studies may not be significantly affected by a wired connection. However, wireless setups such as RF can be bulky and expensive while IR-based options may be limited by line-of-sight restrictions. Recent technological advancements have enabled the development of miniaturised, Bluetooth low energy devices that can be easily integrated with fabricated devices to enable control of drug delivery from mobile devices.^53^

Further progress has also seen the development of closed-loop systems (systems that do not require human intervention).^22,54,55^ These devices can detect when certain conditions are met and can trigger drug delivery autonomously. However, a sensing component is required to monitor ambient conditions. Drug release/delivery is triggered in response to changes in the environment. For example, when a threshold is exceeded. The sensors may monitor for changes in pH, temperature, pressure, or even chemical activity. Implants with this capability may monitor and record electrophysiology signals and use customized algorithms to trigger the delivery of drugs if signals deviate from ‘normal’ or exceed a threshold.

Salam *et al.*,^12^ presented such a device for real-time seizure detection and control intra-cerebrally based on PDMS. They discovered that the seizure onset pattern is heterogenous and recommended a personalised approach to closed-loop system threshold tuning for seizure applications. To resolve individual variations, ‘normal’ or ‘abnormal’ is increasingly defined by machine learning and neural networks which will adapt to each patient. The control signal to trigger is sent *via* an integrated bespoke microprocessor. In the simplest form, platforms such as Arduino and Raspberry Pi can also be used for this. However, in more complex applications, custom Application-Specific Integrated Circuits (ASICs) are designed and fabricated through a foundry.^56,57^

## 3 Sensing

Fluidic-enabled bioelectronic sensors have been widely applied in healthcare and beyond.^58,59^ Microfluidic-based wearables are used extensively for continuous health monitoring, disease detection and sports science.^60–63^ Many different analytes can be detected in a wearable or *in vitro* capacity, with glucose being the most prolific clinically relevant biomarker studied for novel sensing strategies.^64–66^ In addition to the sensing of biomarkers, the use of microfluidic devices for pressure-sensing is abundant.^67–70^ Implantable devices are less common than wearables but there are certain applications where their passive, long-term and high-resolution capability are advantageous.

### 3.1 Microdialysis

**Fig. 2:**
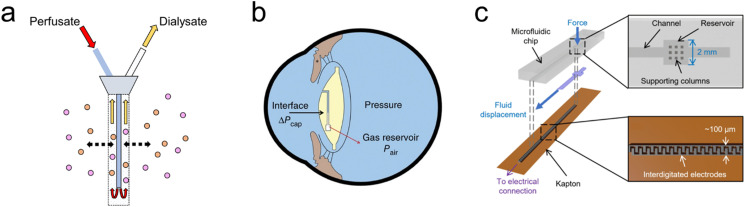
Bioelectronic sensing using microfluidics (a) Microdialysis probe showing the inlet (perfusate) and outlet (dialysate) tubes. The inlet is continuously perfused with an aqueous solution, small solutes then cross the semipermeable membrane by diffusion, the analyte of interest can now be measured at the outlet at regular intervals. (b) Embedded intraocular pressure IOP sensor for pressure measurements to monitor patients with glaucoma. Intraocular fluid enters the sensing channel (circular black line) until equilibrium is reached with the gas inside the reservoir shown, the gas–liquid interface can then be captured by a camera or specially adapted smartphone. (Reproduced with permission from Springer Nature.^76^ Copyright 2014) (c) Microfluidic sensor incorporated into total hip replacement implant for force measurements. When a force is applied, fluid is displaced along a microfluidic channel integrated with electrodes which provide a capacitance readout. (Reproduced with permission.^77^ Copyright 2021 The Authors).

For example, microdialysis is a widely adopted sampling technique in neuroscience, used for the continuous measurement of analyte concentrations in extracellular fluid.^71–73^ A microdialysis probe consists of a semipermeable membrane connected to inlet and outlet tubing, as seen in Fig. 2(a). The inlet is continuously perfused with an aqueous solution (perfusate). Small solutes are then able to cross the semipermeable membrane by passive diffusion. The outlet solution (dialysate) will then contain the analyte of interest and can be collected at certain time intervals. Probe membrane materials include polyacrylonitirile (PAN), polycarbonate-ether, and regenerated cellulose.^74^ While microdialysis remains a key tool for the continual monitoring of neurochemicals within the brain, it is limited by its poor temporal resolution, often on the order of minutes (≥1 min, typically 10 min)^74^ as opposed to the sub-second timescales required for studying neurostimulation events. Continuous online microdialysis seeks to address this issue. For increased time and temporal resolution, an online glucose biosensor and potassium ion-selective electrode were integrated into a microfluidic device to monitor the neurochemical effects of spreading depolarizations (SD).^75^ Concentration changes were successfully monitored in response to SD wave induction, in the range of 10–400 μM with 1 sec time resolution.

### 3.2 Pressure

Fluidic implants have also been engineered to measure pressure in various tissues.^78–80^ A microfluidic-based sensor has been reported in which intraocular pressure (IOP) readings can be made in real-time with a customized optical system by a smartphone camera.^76^ IOP measurements are critical for glaucoma diagnosis and treatment, and are commonly taken in-clinic. However, a snapshot can be misleading given that IOP is highly fluctuating, with normal IOP ranging between 10 and 21 mm Hg.^81^ The sensing mechanism (Fig. 2(b)) is simple and effective, capillary forces and IOP drive liquid into an airtight microfluidic channel, compressing the gas inside the reservoir until gas pressure is in equilibrium with liquid pressure. Increasing the IOP will cause the interface to shift toward the gas reservoir, whereas decreasing the IOP will cause a shift toward the channel opening. Pressure readout is performed through a smartphone camera equipped with an optical adaptor and image analysis software for detection of the aqueous-air interface position. The IOP sensor chip is fabricated using standard lithography out of PDMS with a parylene-c coating to prevent air leakage.

It has already been shown that successful ligament balancing in total knee replacement surgeries rises from 50% to 92–100% using single-use sensor, VERASENSE.^82^ Complications are also reduced by 3.2× if balancing is achieved. This capability in total hip replacement is a major unmet need. A microfluidic enabled bioelectronic pressure sensor has been developed to quantify force feedback within the hip joint, minimising implant failure.^77^ When a force is applied to the fluid reservoir seen in Fig. 2(c), the reservoir deforms and displaces the fluid along the channel. The displaced fluid interacts with the integrated electrodes, increasing the capacitance. On releasing the force, the fluid returns to the reservoir. The sensor consists of a soft elastomeric microfluidic chip layer and a Kapton substrate with aerosol-jet printed integrated electrodes. Sensors are placed at 6 locations within the total hip replacement, representing a powerful research tool to aid implant positioning alongside application to a range of orthopaedic procedures.

## 4 Shape actuation

Soft robotics is a growing field whereby soft materials are used in the development of actuators, creating a controllable, moving system. Fluidic-based soft robotic implants form an exciting capability within novel implantable bioelectronic prostheses. Soft fluidic systems can be used to drive shape actuation of bioelectronic implants, both to reduce invasiveness during implantation and to deliver mechanical stimulation to the body post-implantation. While there is interest in using fluidic-based technology for the continued development of robotic surgical equipment to aid minimally invasive surgery,^83^ there is also emerging interest in converting these actuation techniques for implantable prostheses. Prostheses used to support or replace function in organs ideally would replicate the natural internal shape and material properties to reduce natural immune response. Therefore, the actuation and shape-changing techniques employed by soft-robotic technologies could be a useful translatable tool for the development of implantable prostheses.

### 4.1. Minimally invasive implants

One approach to reducing the invasiveness of surgical implantation of bioelectronic devices is to modify the shape of the device post-implantation. This shape actuation can enable the minimally invasive implantation of large area implants for electrical recording and stimulation of surrounding tissue.^84^ Existing implant technologies, such as an implantable spinal cord stimulator^84^ or a sensing catheter for cardiac mapping,^85^ can be adapted to integrate soft-robotic fabrication techniques and materials. This enables minimally invasive implantation of these devices through shape and size reduction.

**Fig. 3:**
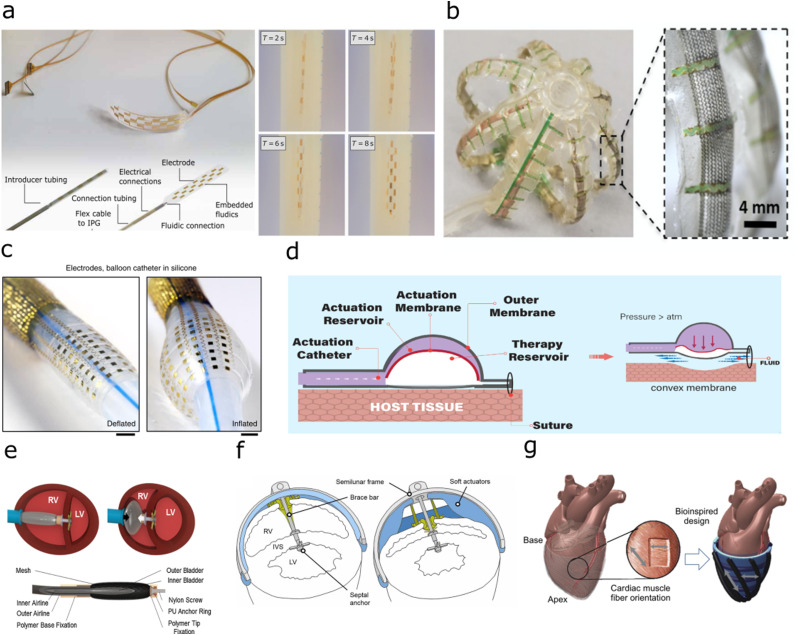
Implants with fluidic shape actuation (a) Minimally invasive spinal cord stimulator using shape actuation to enable deployment using a Tuohy needle before expansion to conform to the spinal. (Reproduced with permission^84^ Copyright 2021 The Authors) (b) Expandable soft robotic sensing arrays for conformable atrial mapping. (Reproduced with permission from AAAS.^85^ Copyright 2020 The Authors, some rights reserved; exclusive licensee AAAS. Distributed under a CC BY-NC 4.0 license) (c) Expandable bioelectronic sensing array fabricated on a silicone balloon catheter. (Reproduced with permission from Springer Nature.^86^ Copyright 2020) (d) Soft robotic mechanical stimulation for foreign body response reduction. (Reproduced with permission from AAAS.^89^ Copyright 2019 American Association for the Advancement of Science) (e) Soft robotic actuator for ventricular ejection. (Reproduced with permission from Springer Nature.^90^ Copyright 2017) (f) Septum-braced soft actuation for ventricular assistance (Reproduced with permission from AAAS.^91^ Copyright 2017 American Association for the Advancement of Science) (g) Soft robotic sleeve providing direct cardiac compression. (Reproduced with permission from AAAS.^92^ Copyright 2017 American Association for the Advancement of Science).

As a key challenge in implantable technologies is the mechanical interface with the surrounding tissue in the body, for optimal interfacing with the soft *in vivo* environment, the implant should ideally mechanically match the surrounding, both in material softness and conformability to the internal surfaces. Fluidic actuation can enable implants that expand to conform to the shape of the target site, enabling higher resolution electrical recording and stimulation (Fig. 3(a)).^84^ Similarly, for implants placed internally within organs, for example inside the heart or blood vessels, the use of fluidic shape actuation enables implants that expand to conform to the internal structure (Fig. 3(b and c)).^85–88^ These types of devices utilise photolithography to fabricate the electrical interface on thin-film biocompatible polymers, such as parylene-c or polyimide.^84,86^ The fluidic actuators are made from thin-film biocompatible polymers, such as silicones and TPU, and can be thermally bonded using laser cutting to weld multiple layers together to form an actuator. Adhesives can then be used to combine the electrical interface with the actuator to form the implant.

Alternatively, the fluidic actuation can enable the material stiffening of a soft implant to aid in implantation, before removal of fluidic pressure to return to the softness of surrounding tissue.^93^ Intriguingly, it has also been reported that by using fluidic actuation of a soft reservoir to deliver mechanical stimulation, the strain and fluid around the implant can be modulated to reduce the foreign body response (Fig. 3(d)).^89^

### 4.2. Mechanical stimulation

The ability of soft robotic devices to shape change through fluidic actuation can enable prostheses that interface with the body to aid in mechanical motion. Externally, as wearable devices, these have been researched for use in mobility aids for joints and muscles.^94^ This ability to aid movement has resulted in translation to implants to aid in movement inside the body. In the cardiac system, current generation ventricular assist devices (VADs) require blood flow to be rerouted to an external pumping system as a therapy for heart failure. Cardiac implants have been developed that utilise soft robotic actuators to provide ventricular assistance, using mechanical stimulation to aid in cardiac compression. Previous work has focussed on two different methodologies, using pneumatic muscles which are implanted into, and braced onto the septum of the heart to directly aid compression in one chamber through the contraction of the cardiac wall (Fig. 3(e and f)),^17,90,91,95^ or a soft-robotic sleeve to provide direct cardiac compression from around the heart (Fig. 3(g)).^92,96^ To form these pneumatically actuated muscles, silicone or thermoplastic elastomer is formed with a metal mesh into a bladder, before adhesive bonding to airline tubing. These types of ventricular assistance can also be triggered with electrical feedback *via* electrocardiogram signals, or pressure-driven feedback measured within the blood vessels to produce a closed-loop assist system to provide tailored therapy to the patient.^97^

## 5 Outlook

The breadth of technologies described in the previous sections illustrate the wide-ranging applications enabled by the convergence of fluidics and bioelectronics. Emerging trends discussed here are still in their early-stage development, with reported demonstrations primarily in pre-clinical models. From drug delivery to sensing to mechanical stimulation, there is vast potential to further mature and develop impactful technologies for healthcare as well as research. Meeting this potential will require overcoming technological and material challenges in several key areas.

### 5.1 Tissue integration

The current generation of fluidic-enabled bioelectronic implants largely consist of polymeric materials with mechanical properties that are not well matched to the surrounding tissues in the body. Improving this mismatch may reduce the foreign body response thereby enabling more effective long-term interventions. For example, for implants into soft tissues such as brain, the integration of softer materials, such as hydrogels could reduce glial scarring and biofouling of critical components. The use of softer and bio-compatible materials could also enable novel, minimally invasive bioelectronic implants that target a wider range of physiological regions. For instance, recording and/or stimulation electrode arrays could be deployed in a minimally invasive manner to the cortical surface for novel brain-machine interfaces, or to the intestine for real-time monitoring of biomarkers for gut health. Device geometry could also be exploited to improve tissue integration. The combination of tissue-compliant materials alongside new fabrication techniques to further miniaturise these classes of devices would enable the targeting of smaller, more delicate regions of clinical interest, for example, cuffs to record and stimulate the peripheral nervous system, or to deliver a high-density electronic array to the retinal surface.

### 5.2 Materials for controlled release and uptake

Development and integration of materials for controlled chemical release or uptake would be particularly helpful for advancing drug delivery and sensing applications. For example, membranes that can change conformation or porosity in response to an applied field.^98^ Modulation of these materials could enable implants that can deliver both charged and non-polar drug molecules to the target site. Selective materials and membranes, such as aptamers or molecularly imprinted polymers could be integrated into implants to enable specific sensing of targeted biomarkers for precise health monitoring. Such active materials would ideally be compatible with established device fabrication processes to streamline integration. For successful translation into the clinic, these materials would also have to match the biocompatibility requirements of implantable devices.

### 5.3 Control systems

A critical challenge in the clinical translatability of using shape actuation-based implants, as well as some drug and sensing devices, is the fluidic pump and control systems required to drive implantable devices. In the research phase of development, these systems are often connected to external pumping systems, but in some cases, the pump would ideally be implanted simultaneously inside the body, eliminating any external connections which could be a source of infection. Hence, there is a need to develop miniaturised and implantable fluidic pumping systems that can provide the fluidic pressures and flow rates required for therapy. These miniaturised pumps would ideally incorporate soft materials, such as silicones and/or hydrogels, for better tissue integration. As multifunctional devices with dual capacity for sensing and therapy mature, future control systems may leverage developments in machine learning and closed-loop actuation to realize autonomous and semi-autonomous systems that can tailor therapy (*e.g.* drug release or mechanical stimulation) in response to determinations from real-time monitoring of appropriate biomarkers.

### 5.4 Connections

Electrical and fluidic connections and interconnects are a potential failure point in fluidic-enabled bioelectronic implants. Connections and interconnects must be able to withstand the mechanical, biological and electrochemical stresses of implantation procedures and use inside the body. Developments in novel bioelectronic implants would benefit from complementary developments in robust and scalable strategies for electrical and fluidic connections to external recording and control systems. In some cases, integration of wireless technologies for both power and data transfer could enable these devices to be implanted without external interfacing. For example, pressure sensors could be particularly suitable for wireless readouts. Similarly, wireless and/or multiplexed electrical connectivity could enable higher channel counts for stimulation and recording of the target tissue, increasing the electrical resolution of these implants. For applications that require hardwired connections, further advances in connection strategies, including novel biocompatible adhesives^99^ and anisotropic conducting materials,^100^ could facilitate the development of new applications as well as translation to the clinic.

### 5.5 Validation for translation

As fluidic-enabled bioelectronics mature, promising technologies should be coupled to development pathways for (clinical) translation. To date, the technologies discussed in this article have primarily been tested in relatively early-stage pre-clinical models including acute and short-term tests in rodents, large animals and human cadavers. Translation to clinical use will require further validation in appropriate models, including in most cases, demonstration of long-term safety and efficacy *in vivo*. The use of advanced *ex vivo*^84^ as well as *in vitro* models for accelerated aging^101^ may accelerate identification of failure mechanisms and other technological challenges prior to such *in vivo* studies. Additional biocompatibility testing is likely to be required particularly for implants that incorporate novel materials. Finally, as a steppingstone to the clinic, fluidic-enabled bioelectronic implants may find utility as pre-clinical research tools which in turn could ultimately lead to the discovery of new therapies while also de-risking subsequent clinical translation of related technologies.

## Data access

This is a perspective article and generated no new data. All data underlying this study are cited in references.

## Conflicts of interest

There are no conflicts to declare.

## Supplementary Material
